# A Noise-Aware Coding Scheme for Texture Classification

**DOI:** 10.3390/s110808028

**Published:** 2011-08-15

**Authors:** Mohammad Shoyaib, M. Abdullah-Al-Wadud, Oksam Chae

**Affiliations:** 1 Department of Computer Engineering, Kyung Hee University, Yongin 446-701, Korea; E-Mail: shoyaib@khu.ac.kr; 2 Department of Industrial and Management Engineering, Hankuk University of Foreign Studies, Yongin 449-791, Korea; E-Mail: wadud@hufs.ac.kr

**Keywords:** noise tolerant ternary pattern, adaptive noise band, texlet

## Abstract

Texture-based analysis of images is a very common and much discussed issue in the fields of computer vision and image processing. Several methods have already been proposed to codify texture micro-patterns (texlets) in images. Most of these methods perform well when a given image is noise-free, but real world images contain different types of signal-independent as well as signal-dependent noises originated from different sources, even from the camera sensor itself. Hence, it is necessary to differentiate false textures appearing due to the noises, and thus, to achieve a reliable representation of texlets. In this proposal, we define an adaptive noise band (ANB) to approximate the amount of noise contamination around a pixel up to a certain extent. Based on this ANB, we generate reliable codes named noise tolerant ternary pattern (NTTP) to represent the texlets in an image. Extensive experiments on several datasets from renowned texture databases, such as the Outex and the Brodatz database, show that NTTP performs much better than the state-of-the-art methods.

## 1. Introduction

Noise is an inherent property of images and becomes a major obstacle in detecting texture patterns. Different types of noises might originate from different sources. For example, photons coming from an object undergo a series of processing, and finally form a pixel in a CCD/CMOS sensor based digital camera, where there may be a possibility of inclusion of different types of noises in every stage, such as photon shot noise (signal dependent noise) and readout noise (signal independent noise).

Over the last few decades several proposals have been proposed for detecting texture micro-patterns. Among them, Gabor wavelet [1,2] and local binary pattern (LBP) [3] based methods have become popular for their competitive accuracies. Gabor features are obtained by a 2-D Gabor wavelet transformation of the grayscale image. A family of Gabor kernels (usually eight different orientations and five different scales) is convolved with an image to extract micro-patterns, such as lines and edges, in different scales and orientations. The statistics of such micro-patterns can be used to describe the underlying textures in the image, and has been used for different types of image based applications [4–8]. However, the cost of convolving an image with the Gabor kernels is very high as the expensive convolution process needs to be performed several times for each pixel. Furthermore, miniaturizing hand-held devices demands computation of different applications with fewer resources (e.g., CPU and memory) while maintaining higher accuracy. Thus Gabor-based methods may not be suitable for many devices. LBP, which is calculated simply by comparing a pixel with its neighbors, has gained popularity for its computational simplicity and robustness against monotonic changes in illumination. It has already shown its capabilities in several image processing applications such as face detection [9], face recognition [10], facial expression detection [11], moving object detection [12], finger biometric recognition [13] and many others [14–16]. However, LBP is very noise sensitive, especially in near uniform regions. Small changes in intensities due to noises in near uniform regions may lead to erroneous LBP codes (Example 1 in Section 3.2 presents one such example). Thus even for small intensity fluctuations, which are very common in digital images, LBP and most of its variants fail to generate the same code for the same type of texture structures. Keeping this in mind, Tan and Triggs proposed Local Ternary Pattern (LTP) [10], which uses three-value encoding and shows tolerance to noise up to a certain level as they assume noises in an image usually vary within a fixed threshold (±5). However, the amount of noise may vary with the intensity level of pixels in an image [17–19]. Thus the fixed threshold may fail to deal with different types of noises, especially the signal dependent noises. Hence, different thresholds may be required to deal with the presence of different extent of noises in an image. Furthermore, LTP uses difference of Gaussian (DoG) filtering as a preprocessing step, which is a band pass filtering used to reduce, for instance, aliasing and noise effects. However, this expensive filtering may also eliminate some important texlets, which might aid the classification scheme. Recently, a few promising variants of LTP have also been proposed, focusing mainly on the medical application domain. For example, elongated quinary pattern (EQP) [20], dominant local ternary pattern (DLTP) [21] and extended local ternary pattern (ELTP) [22]. Among them, EQP uses five-value encoding to handle noises, however increasing the number of quantization levels also increase the size of the feature vector and computational demands. Further, gradient filtered images are used to calculate ELTP features. However, these variants are mostly proposed targeting on specific domains. Hence, they may not always fit for general purpose texture detection.

To get rid of the curse of sensitivity of LBP code due to noise, LBP variance (LBPV) [23] follows a tricky way where it uses local variance information to suppress the effects of inconsistent LBP code in near uniform regions and magnifies the effects of LBP code calculated in highly textured regions. However, as long as the original code is not reliable, such suppression and magnification based on local variances might mislead the classification. The reason is that it might suppress (or magnify) the contribution of textures important (or unimportant) for classification. Further, the variances may change with the change of image contrast. This may lead to different values in feature vectors for similar textures with different contrast, which may affect the performance. Recently, Completed Local Binary Pattern (CLBP) [24] considers both the signs and the magnitudes of the differences between a pixel and its neighbors, and it also incorporates the pixel information itself. Thus, in CLBP, the incorporation of magnitude information with sign may help for retaining huge information to describe texlets for classification. However, combining such different information make the size of feature vector very large, which increases computational time and memory requirements. Moreover, though the use of magnitude along with sign information may implicitly handle some noisy fluctuations in intensities, it does not offer any effective mechanism of handling different noises while generating the texlet codes. This may hamper the performance.

To solve the aforementioned problems, we first introduce an adaptive noise-band (ANB) to provide an estimate of noise within certain limits. The ANB is then used to generate a noise tolerant ternary pattern (NTTP) at every pixel in an image. Finally, the NTTP codes are used to form a feature vector to describe an image, which possesses the desired characteristics for texture classification, namely:

- simplicity in computation, and
- robustness against common noises appearing in digital images.

All these properties of the proposed NTTP lead to a better accuracy in texture classification than the state-of the-the-art methods. The rest of this paper is organized al follows. Section 2 briefly describes some well-known state-of-the-art methods, the proposed method is illustrated in Section 3, experimental results along with related discussion are presented in Section 4, and finally Section 5 concludes the paper.

## 2. A Brief Overview on Existing Feature Descriptors

In this section, we briefly describe four state-of-the-art methods, namely local binary pattern, local ternary pattern, local binary pattern with variance, completed local binary pattern. Besides these methods, we also briefly discuss dominant local binary pattern along with a few LTP-based methods.

### 2.1. Local Binary Pattern

**Figure 1.**
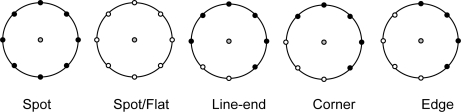
Examples of the texlets encoded by LBP.

Local Binary Pattern (LBP) [3] is an *n*-bit binary code at a pixel, *c*, in a grayscale image is generated by Equation (1), which compares *c*’s intensity with that of its *n* neighbors. These neighbors are located at uniform distances on a circle centered at *c* with radius *r*:

(1) $${\mathrm{LBP}}_{n,r}(x_{c},y_{c})=\sum_{l=0}^{n-1}q(g_{1}-g_{c})2^{l},\;\;q(a)=\{\begin{array}{cc} 1 & \text{if }\;\;\;\;a\geq 0\\ 0 & \text{otherwise} \end{array}$$

where (*x_c_*, *y_c_*) is the pixel co-ordinate of *c*, *g_c_* and *g_l_* are the intensities of *c* and the *l*^th^ neighboring pixel, respectively. The LBP codes can represent texlets such as edge, corner and line-end. Figure 1 presents such patterns.

Usually, *n* ∈ {4,8,16,24} and *r* ∈ {1,2,3,4} are used for the calculation of LBP. Increasing the number of neighbors, *n*, in LBP code helps to incorporate more spatial information. However, this grows the size of the code, which increases the computational as well as space complexity. Using such LBP codes of an image, a histogram is built to represent the feature vector of that image. Equation (2) derives the *k*^th^ component of the histogram of an image of size *M* × *N*:

(2) $$H^{k}=\sum_{i=0}^{M-1}\sum_{j=0}^{N-1}\psi_{i,j}^{k},\;\psi_{i,j}^{k}=\{\begin{array}{cc} 1 & \text{if}\;{\mathrm{LBP}}_{n,r}(i,j)=k\\ 0 & \text{Otherwise} \end{array}$$

### 2.2. Uniform Local Binary Pattern

An LBP is defined as uniform local binary pattern (ULBP) if there are at most two bit transitions in its binary equivalent [25]. In other words, for a uniform pattern, the value of U(·) in Equation (3) can be at most 2:

(3) $$U({\text{LBP}}_{n,r}(x_{c},y_{c}))=|q(g_{n-1}-g_{c})-q(g_{0}-g_{c})|+\sum_{l=0}^{n-1}\left|q(g_{l}-g_{c})-q(g_{l-1}-g_{c})\right|$$

For example, 11100011 is a uniform pattern, while 11101011 is not. When uniformity is taken into consideration, all the non-uniform patterns are accumulated in a single bin during histogram formation. With *n* = 2, there are 58 different uniform patterns, and hence the histogram will contain 59 bins in total.

### 2.3. Local Ternary Pattern

Local Ternary Pattern (LTP) [10] mainly follows the same spirit of LBP. The key difference is that it introduces a new bit to manage the intensity fluctuations. Thus, LTP becomes a ternary code at a pixel *c*, which is generated by Equation (4):

(4) $$\mathrm{LT}P_{n,r}(x_{c},y_{c})=\sum_{l=0}^{n-1}q(g_{l}-g_{c})3^{l},\;\;\;\;\;\;\;\;q(a)=\{\begin{array}{cc} 1 & \text{if }a\geq \alpha \\ -1 & \text{if }a\leq -\alpha \\ 0 & \text{Otherwise} \end{array}$$

Here, the value of α is set to 5. To reduce the size of the feature vector, an LTP code is usually split into two binary codes (upper pattern and lower pattern). For an image, two histograms are built separately for the two types of codes to represent the feature vector of that image. Tan *et al*. [10] also propose to perform some pre-processing before the code generation, such as difference of Gaussian filtering (DoG) filtering, gamma correction, illumination normalization and masking.

### 2.4. Local Binary Pattern with Variance

The methodology for code generation of LBPV [23] is same as LBP. The only difference is the way of calculating the histogram (Equation (6)), where it incorporates local variance (Equation (5)) as a weight *w_i,j_*:

(5) $$w_{i,j}=\frac{1}{n}\sum_{l=0}^{n-1}{\left(g_{l}-\frac{1}{n}\sum_{m=0}^{n-1}g_{m}\right)}^{2}$$

(6) $$H^{k}=\sum_{i=0}^{M-1}\sum_{j=0}^{N-1}w_{i,j}\psi_{i,j}^{k}$$

### 2.5. Completed Local Binary Pattern

Completed local binary pattern (CLBP) [24] consists of three components: a code generated using only the sign value of the differences between a pixel and its neighbors (CLBP_S), a code using the magnitudes of those differences (CLBP_M) and a code from center pixel’s intensity with respect to the average image intensity (CLBP_C). The generation procedure of CLBP_S is exactly same as LBP. CLBP_M is generated following Equation (7):

(7) $$\mathrm{CLBP}\_M_{n,r}(x_{c},y_{c})=\sum_{l=0}^{n-1}p(|g_{l}-g_{c}|,t)2^{l},\;\;\;\;p(z,t)=\{\begin{array}{cc} 1 & \text{if}\;z\geq t\\ 0 & \text{otherwise} \end{array}$$

where *t* is the mean of all |*g_l_* – *g_c_*| values in the whole image.

The CLBP_C is coded as:

(8) $$\mathrm{CLBP}\_C(x_{c},y_{c})=p(g_{c},t_{I}),\;\;\;\;p(z,t_{I})=\{\begin{array}{cc} 1 & \text{if }\;z\geq t_{I}\\ 0 & \text{otherwise} \end{array}$$

where *t_I_* is the average gray level of the whole image.

These three codes (CLBP_C, CLBP_S and CLBP_M) can be combined in one of two ways. The first way is by building a joint 3D histogram, which is denoted by CLBP_S/M/C, and the second way is to: (i) build a 2-D joint histogram using CLBP_S (or CLBP_M) and CLBP_C, denoted as CLBP_S/C (or CLBP_M/C), (ii) convert this 2-D histogram into a 1-D histogram, and then (iii) concatenate CLBP_M (or CLBP_S) to generate a joint histogram denoted by CLBP_M_S/C (or CLBP_S_M/C).

### 2.6. Dominant Local Binary Pattern and some LTP Variants

To cope up with the changes of LBP codes due to noisy fluctuations, dominant local binary pattern (DLBP) [26] combines the strength of both LBP and the Gabor filter. Here only the most frequently occurring LBP patterns are used, which are assumed to provide the descriptive textural information. On the other hand, Gabor-based features supply global textural information. Thus the combination of both frequent LBP codes and Gabor features is expected to perform well.

Following DLBP, Nanni *et al*. [21] proposed to select histogram bins with higher variance from the original LBP and LTP patterns as dominant patterns. In [20], instead of three levels of quantization as in LTP, the authors analyzed more levels of quantization along with different shapes of neighborhoods information, and concluded that an elliptical shape neighborhood with five level quantization works well. For duodenal texture classification, an adaptive LTP is also proposed in [22], where the thresholds are determined based on a weighted combination of training and observed data.

## 3. Proposed Methodology

The proposed method comprises with two basic parts: noise band approximation based on the observed signal and generation of a texture code with the aid of this band. In this section, we first introduce the noise band estimation methodology, and then present the proposed NTTP coding scheme.

### 3.1. Adaptive Noise Band

When a digital image is captured using CCD/CMOS sensors, images might be contaminated with different types of noises, for instance, photon shot noise, dark current noise, read noise, thermal noise and quantization noise [27]. Thus an observed signal, *g*(*x*), can be written as the combination of noise free original signal, *f*(*x*), and the noise, *δ*(*x*), as follows:

(9) g(x)=f(x)+δ(x)

Here, *δ*(*x*) may represent signal dependent or independent noise, or a combination of them [17,18]. In this proposal, we focus on the approximation of an upper bound of the resulting noise *δ*(*x*) at a given pixel when the extent of noise in the image is within certain limit (if the noise contamination is too high, then special treatment is required, which is beyond the scope of this paper). Such an approximation of noise helps to differentiate between actual texture patterns and noisy fluctuations around a pixel. For considerable values of signal-to-noise-ratio (SNR) around a pixel, we propose that the extent of *δ*(*x*) can be approximated by taking the square root of the observed signal values, *g*(*x*). Lemma 1 justifies this approximation.

**Lemma 1:** $\sqrt{g(x)}\geq \delta (x)$ when SNR is higher than a certain value.**Proof:** Let $\gamma =\frac{f(x)}{\delta (x)}$.Now:

$$\begin{array}{l} \sqrt{g(x)}\geq \delta (x)\\ \Rightarrow \sqrt{f(x)+\delta (x)}\geq \delta (x)\\ \Rightarrow \sqrt{\gamma \delta (x)+\delta (x)}\geq \delta (x)\\ \Rightarrow \delta^{2}(x)-\gamma \delta (x)-\delta (x)\leq 0\\ \Rightarrow \delta (x)\leq \gamma +1. \end{array}$$

Replacing *δ*(*x*) by $\frac{f(x)}{\gamma }$ yields:

(10) $$f(x)\leq \gamma^{2}+\gamma$$

Thus *γ* sets a limit on *f*(*x*) for $\sqrt{g(x)}\geq \delta (x)$ to be true. For example, when *γ* = 15, the condition will be true when *f*(*x*) ≤ 241. Again, from Equation (10), we get:

(11) $$\left(\gamma -\frac{-1+\sqrt{1+4f(x)}}{2}\right)\left(\gamma -\frac{-1-\sqrt{1+4f(x)}}{2}\right)\geq 0$$

Equation (11) gives the lower bound on *γ* as:

(12) $$\gamma \geq \frac{-1+\sqrt{1+4f(x)}}{2}$$

To cover the grayscale values of the whole dynamic range, *i.e*., [0, 255], we put *f*(*x*) = 255 in Equation (12), which yields *γ* ≥ 15.47655. Hence, the lemma holds.

Lemma 1 gives a lower bound on *f*(*x*)/*δ*(*x*) to be higher than 15.48. Such a condition is usually fulfilled by digital images if there is not too much outer world noise. We can, therefore, approximate the possible contamination of noises for a given pixel by taking the square-root value of that pixel, and thus we define the adaptive noise band (ANB) in Definition 1.

**Definition 1:** *Adaptive noise band (ANB)*

An adaptive noise band defines the maximum possible extent of noise that can be contaminated with the observed intensity, *i*, of a pixel, and can be approximated by the square root of that intensity.

When the difference between two neighboring pixel values is within the ANB, we consider that the difference is due to noise. In some pixels, the ANB may results in inclusion of orignial texture patterns into the noise band. However, this may not casuse much harm to the classification; rather this helps to distinguish prominent and less prominent patterns, which results in improved classification.

**Figure 2.**
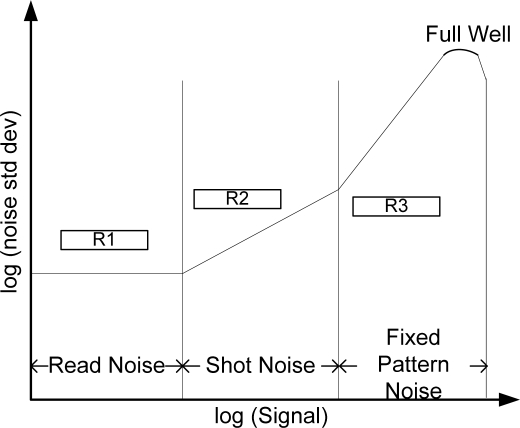
A photon transfer curve for CCD output signals.

Analyzing the Photon Transfer Curve (PTC) [28] presented in Figure 2, we find that the noise characteristics do not remain same for different intensity region. For low intensity region (R1), the dominant noises are independent of signal and remain constant. Another point here is that this PTC curve is calculated in the sensor domain (light space), but our focus is to deal with noises in the given image (image space). This problem is addressed by Faraji *et al.* in [17], where it is shown that this constant behavior is also observed in the image space. Since the gray levels at this region are low, the noises are usually higher than the square root of the observed intensity. We, therefore, slightly modify the ANB at intensity *i* as:

(13) $$\mathrm{AN}B_{i}=\{\begin{array}{cc} \pm \tau & \text{if}\;i<R\\ \pm \sqrt{i} & \text{otherwise} \end{array}$$

where *R* is the boundary point separating the low-intensity region in image space that corresponds to the R1 region in Figure 2, and *τ* is a constant to be set empirically. The value of *R* can be found in different ways. According to the method in [17], the value of *R* can be picked from the plot of intensity *vs*. noise standard deviation. In such plots, the breaking points in the curve represent different noise regions. Beside this, since noise remains constant for the low intensity regions, we can have the amount of noise in this region by calculating the average of the standard deviation of pixel intensities in some homogenous patches in the given image. This gives the horizontal line indicating the constant noise. Again according to [29], skellam parameters maintain a linear relationship with the pixel intensities, and the signal dependent noises (SDN) can be calculated from the skellam parameters. Thus we can approximate another line from the plot of intensity *vs*. SDN. The intersecting point of these two lines gives the value of R.

### 3.2. The Proposed Texture Descriptor

The proposed NTTP uses the aforementioned ANB to encode the texture pattern for a given pixel, where the NTTP code at a pixel *c*, coordinated at (*x_c_,y_c_*) is generated by comparing its intensity *g_c_* with its neighbors according to Equation (14):

(14) $$\mathrm{NTT}P_{n,r}(x_{c},y_{c})=\sum_{l=0}^{n-1}q(g_{l}-g_{c})3^{l},\;\;\;\;q(a)=\{\begin{array}{cc} 1 & \text{if}\;a\geq {\text{ANB}}_{g_{c}}\\ -1 & \text{if }a\leq -{\mathrm{ANB}}_{g_{c}}\\ 0 & \text{Otherwise} \end{array}$$

where *n* is the predefined number of neighbors at equal distance on a circle of radius *r* centered at (*x_c_*, *y_c_*). Here ANB helps to distinguish the fluctuation of intensities due to noises, which in turn helps to generate a reliable code for texture description.

According to Equation (14), the total number of different possible *NTTP_n,r_* codes is 3*^n^*, which is huge and computationally not feasible to use. To handle it, we split a *NTTP_n,r_* code into two separate binary codes: an upper (*NTTP_U_n,r_*) and a lower (*NTTP_L_n,r_*) code by modifying Equation (14) as follows:

(15) $$\begin{array}{l} \mathrm{NTTP}\_U_{n,r}(x_{c},y_{c})=\sum_{l=0}^{n-1}q(g_{l}-g_{c})2^{l},\;\;\;q(a)=\{\begin{array}{cc} 1 & \text{if}\;a\geq \mathrm{AN}B_{g_{c}}\\ 0 & \text{Otherwise} \end{array}\\ \mathrm{NTTP}\_L_{n,r}(x_{c},y_{c})=\sum_{l=0}^{n-1}q(g_{l}-g_{c})2^{l},\;\;\;q(a)=\{\begin{array}{cc} 1 & \text{if}\;a\leq \mathrm{AN}B_{g_{c}}\\ 0 & \text{Otherwise} \end{array} \end{array}$$

**Figure 3.**
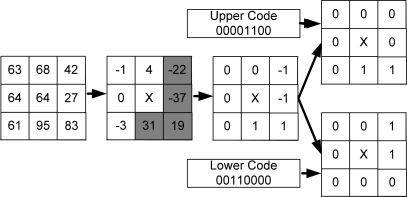
Conversion of a ternary pattern into two equivalent binary patterns used in NTTP.

An example of such splitting is demonstrated in Figure 3 using *n* = 8 and *r* = 1. It is noteworthy to point here that we can get back the original ternary code by combining (performing logical XOR and using sign information) these two binary codes. So, such decomposition is lossless. Example 1 presents the robustness of *NTTP* over LBP and LTP, which represents the capability of handling noisy fluctuations and thus helps to generate stable code in different situations.

**Figure 4.**
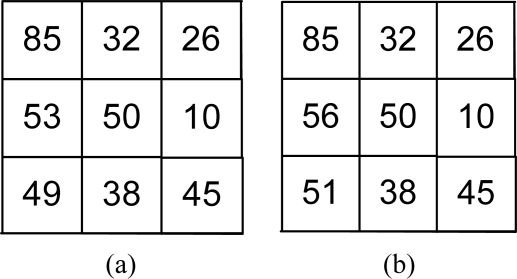
Change of intensity due to noisy fluctuations. (**a**) Original pattern; (**b**) The pattern when two pixel values are changed due to noisy fluctuations.

**Example 1:** Let us consider that Figure 4(a) shows a texture pattern around the pixel having intensity 50 in an image, and Figure 4(b) shows the same pattern but the intensities 49 and 53 are changed to 51 and 56, respectively, due to noisy fluctuations.

Here LBP cannot handle the change in intensity 49 as the corresponding bit (calculated by comparing it with 50 in the LBP) changes from 0 to 1 when the pixel value becomes 51. LTP is robust against the fluctuation of the intensity from 49 to 51 since both values will lead to 0 in the corresponding bit of the LTP code because of the threshold *α* = 5 in Equation (4). However, LTP fails to handle the fluctuation from 53 to 56, where the corresponding bit changes from 0 to 1. In the proposed NTTP, *ANB*_50_ ≈ 7, and hence it can generate the same code in spite of the presence of these intensity fluctuations.

As we have discussed so far, the NTTP considers only the comparisons between a pixel and its neighbors. However, the grayscale of the center pixel also possesses some meaningful information, which is important for classification [30]. Hence, following [24], we codify the center pixel’s grayscale value, *g_c_*, with respect to the whole image using Equation (16), and represent it as *NTTP_C_n,r_*:

(16) $$\mathrm{NTT}P_{C_{n,r}}(x_{c},y_{c})=p(g_{c},t_{I}),\;\;\;p(z,t_{I})=\{\begin{array}{cc} 1 & \text{if}\;z\geq t_{I}\\ 0 & \text{otherwise} \end{array}$$

where *t_I_* is the mean gray level of the whole image.

To describe the pattern in an image, we only consider uniform patterns (described in Section 2.2) for both upper and lower NTTP codes. This results in a much reduced sized feature vector. To build the feature vector, we first calculate two 2-D histograms using NTTP_U and NTTP_C, and NTTP_L and NTTP_C. We then convert each of these 2-D histograms into a 1-D histogram, and then concatenate them to build the final NTTP feature vector.

Algorithm 1 presents a picture of whole procedure for NTTP feature vector generation, which is simple and easy to calculate. In comparison with LTP, the added computational complexity is the calculation of *ANB* and the incorporation of the center pixel information, which is linear time process.

**Algorithm 1.** Extraction of NTTP feature vector of an image.

**Input:** An image *I* and parameters *n*, *r*, *τ* and R.
**Output:** The NTTP feature vector of *I.*
Initialize all the entries in two 2-D histograms *H_CU_* and *H_CL_* to zero. **For** each center pixel do Calculate *NTTP_U_n,r_* and *NTTP_L_n,r_* according to Equation (15) Calculate *NTTP_C_n,r_* according to Equation (16) Assign the uniform code indices of *NTTP_U_n,r_* and *NTTP_L_n,r_* to U and L, respectively Increase the corresponding bins *H_CU_*[*NTTP_C_n,r_*][*U*] and *H_CL_*[*NTTP_C_n,r_*][*L*] by 1 **End For** Convert both *H_CU_* and *H_CL_* into 1-D histograms and concatenate them to form a 1-D histogram *H* Return *H*

## 4. Experimental Results and Discussion

In this section, we first describe the evaluation protocol used in our experiments that includes the sources and descriptions of the experimental datasets (in Section 4.2) as well as the criteria to evaluate the performances of different methods (in Section 4.2), and then we present the experimental results (in Section 4.3).

### 4.1. Source of Experimental Data

**Figure 5.**
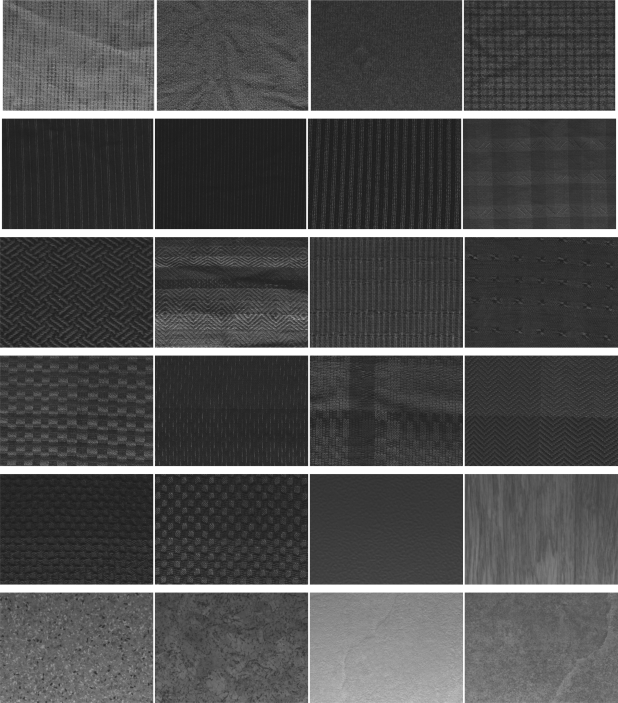
Sample 24 texture images in the Outex texture database.

We use the well-known Outex texture database [31] to compare the performance of NTTP with four state-of-the-art methods, namely LBP, LBPV, CLBP and LTP. Among several datasets available within this database, we use the first ten datasets for our experiments, namely Outex_TC_00000 (TC00), Outex_TC_00001 (TC01), Outex_TC_00002 (TC02), Outex_TC_00003 (TC03), Outex_TC_00004 (TC04), Outex_TC_00005 (TC05), Outex_TC_00006 (TC06), Outex_TC_00007 (TC07), Outex_TC_00008 (TC08) and Outex_TC_00009 (TC09). For all the experiments, we use the same setup for training and test data that is provided along with the database unless otherwise specified. Each of these dataset includes 24 different textures as displayed in Figure 5. The datasets differ in the number of training and test images and the size of the images. Different sizes of windows are used to crop different sizes of images from the 24 source images. For example, for the windows of sizes 128 × 128, 64 × 64 and 32 × 32, total 20, 88 and 368 images, respectively, can be cropped from a given source image. The cropped images are randomly divided into two halves of equal sizes to have the training and test sets. Such division ensures an unbiased performance estimate. Furthermore, for a given test suite, this random partitioning is repeated 100 times, resulting in a test suite of 100 individual problems, for each of the ten datasets, which offers more reliable performance evaluation.

We also use the Brodatz database (available online: http://www.outex.oulu.fi/index.php?page=contributed; contrib_TC_00004 dataset) [32,33], which consists of 32 different textures, to show the robustness of the proposed NTTP coding scheme. Even though the Outex is one of the more appropriate databases for the evaluation of our method (because the images are taken here under sensor based camera and they provide reference background images) we choose the Brodatz database as it is the mostly used benchmark database. The images are 256 × 256 in size with 256 gray levels. From these images, a total of 2,048 images of size 64 × 64 pixels are cropped, where there are 64 samples for each of the 32 texture classes. We apply a 10-fold cross validation on this dataset, which randomly partitions the dataset into 10 parts and generates 10 different problem sets considering nine parts as training and the other part as test set.

### 4.2. Classification and Evaluation Protocol

In the literature several classifiers are available, whose performance varies in different data and applications. However, in this paper our goal is to show the relative performances of different texture descriptors. So, without looking for best suited classifier relevant with our dataset, we adopt two well known classifiers in our experiments. The first one is the well known the nearest neighbor classifier using simple chi-square distance (Equation (17)). Here, for a given feature vector (*X*) of a texture image, we find the distances from all the known models (feature vectors), and assign it to the class whose model (*Y*) gives the minimum distance:

(17) $$D(X,Y)=\sum_{i=0}^{L-1}\frac{{\left(X_{i}-Y_{i}\right)}^{2}}{\left(X_{i}+Y_{i}\right)}$$

where *L* is the size of the feature vector.

We also apply support vector machine (SVM) [34] to classify the textures, and to test the performances of the different feature vectors in machine learning algorithms. For this, we follow the one-against-one strategy and use the library of SVM described in [35]. We use simple percentage of correct detections as the evaluation protocol for generating different results in our experiments.

### 4.3. Result and Discussion

The main goal of this proposal is to achieve a robust coding scheme, which is able to generate same code for same texture irrespective of the presence of noisy fluctuations of intensities, for texture based analysis of images. To show the robustness of the proposed NTTP coding scheme as compared to the state-of-the-art methods, we keep all the parameters the same and compare the basic coding schemes only. In our experimentations, we use *n* = 8 and *r* = 2, and uniform pattern (except CLBP, as the original proposal does not include uniform pattern) for all the methods. Further, using some homogenous patches and skellam parameters we approximate the value of R as 26. In our experiments, we set *τ* = 5 following [10] as this value works well for low intensity region. No preprocessing is performed on the images unless stated otherwise.

The official website (http://www.outex.oulu.fi/index.php?page=classification) of the Outex Database maintains the best results (BR) to date based on the submission of different authors. We incorporate these results from the website in Table 1. Moreover, we include the results of Gabor filter on the same datasets from [31]. Besides these two best results (from website and from [31]), we calculate the average results over the 100 test sets from each dataset for the four state-of-the-art methods and for the NTTP. Table 1 summarizes the performances.

In case of *LTP*(pre), we use the preprocessing proposed in [10]. As mentioned earlier, such preprocessing, especially the expensive DoG filtering, may filter out some important micropatterns and degrade the results. For this reason, *LTP* without preprocessing performs better. Again the main reason behind the better performances of LTP in many cases is its fixed noise band. This band helps it to manage some noisy intensity fluctuations during the texture codification. On the other hand, the NTTP shows excellent performance as compared to the others in every case. This is mainly achieved by using adaptive noise band. Further, the influence of the center pixel information also increases the overall performances.

In CLBP, there are three different proposals. In Table 1, we only include the results of the best performing proposal, denoted as *CLBP*_*S/M/C*. However, its performances are even comparable with that of the basic LBP proposal in many cases, which is demonstrated in Table 1. This is because, like LBP, CLBP is also sensitive to near uniform region. By including three different types of information such as sign, magnitude and center pixel information, the patterns due to the fluctuation of intensities (caused by noises) are expected to result in scattered accumulation in different histogram bins while the prominent patterns may contribute to some specific bins. Therefore, the noisy patterns are assumed to have less effect on the performance. However, this may not be always true because both noisy and actual patterns may contribute to the same bins, which may lead to poor performance as it is shown in Table 1. LBPV gives higher weights for the codes in high variance regions. This is because it is generally assumed that high variance area has better discrimination power. However, codes from low/moderate variance area may have good contributions for classification. Thus, the performances of LBPV become even lower then LBP in many cases.

To check the reliability and the stability of the codes, we further consider four difficult datasets such as TC02, TC05, TC07 and TC09, where the performances of all the methods are relatively lower according to the Table 1. In this experiment, we calculate the mean, minimum, maximum and standard deviation of the accuracies over the 100 test sets from each dataset for all the methods. Table 2 shows the results of this experiment for our method along with the sate-of-the-art methods. It is observed from the Table 2 is that the NTTP achieves not only the highest accuracies but the differences between the maximum and minimum accuracies as well as the standard deviation of the results are also the lowest. This advocates for the better reliability of NTTP than the other methods.

For observing the robustness of different methods when extra additive noises are added to the images, we add white Gaussian noise (WGN) to the test images in the TC02 dataset. Such an experimental setup helps to identify the strengths of different coding schemes under noisy environments. Using the chi-square distance and the nearest neighbor classifier, the correct detection rates of LBP, LBPV, CLBP_S/M/C, LTP and NTTP are found as 65.38%, 73.56%, 72.96%, 76%, 87.44%, respectively, which also show the better noise tolerance of the proposed method as compared to other methods.

In addition to the chi-square distance along with the nearest neighbor classifier, we also use the well known machine learning approach, SVM, to test the performances of different methods. In this experiment, we use radial basis function (RBF) as kernel, and TC02 and TC09 datasets from the Outex database. In each case, we perform a 10-fold cross validation test, and the averages of the accuracies are presented in Table 3. It shows that using SVM, we get better accuracies than the nearest neighbor classifier for all the pattern codes. However, the proposed NTTP shows better performances over the other existing coding mechanisms.

Finally, to test the consistency of the codes, we apply both of our adopted classifiers on a dataset collected from the well known Brodatz database. The average of the 10-fold cross validation results are presented in Table 4. Here, the performances of all the methods are relatively better as compared to those obtained for the Outex database. However, NTTP shows better results compare to others in this dataset too.

## 5. Conclusions

In this paper, we propose NTTP to encode the texlets in images. It is computationally simple, but robust against the noises that generally appear in images. This robustness is achieved by dint of using an adaptive noise band (ANB) that is capable of managing, up to a certain extent, different noises like the sensor/camera noises. Thus the proposed NTTP can be used for different kind of texture based classification like face recognition, facial expression recognition, content based image analysis and several different applications in the presence of common noises in the images. In our proposal, the ANB approximates noises based on single pixel information only. However, it may fail to handle very a large extent of noise contamination. Incorporating more information (such as neighboring pixel information) and/or some filtering process with our coding scheme might help in this regard. We leave it here as our future work.

## Figures and Tables

**Table 1.** Classification rates (%), using chi-square distance and nearest neighbor classifier, of the state-of-the-art methods and the proposed method.

	**TC00**	**TC01**	**TC02**	**TC03**	**TC04**	**TC05**	**TC06**	**TC07**	**TC08**	**TC09**
LBP	99.58	97.76	84.21	99.15	97.76	83.80	97.93	87.34	97.87	87.43
CLBP_S/M/C	99.16	98.52	86.17	99.14	98.51	86.23	98.13	88.13	98.17	88.30
LTP(pre)	99.16	96.40	76.55	99.35	96.45	74.92	96.42	82.26	96.58	81.65
LTP	99.50	97.40	90.00	99.61	99.12	90.01	98.96	91.52	98.95	91.67
LBPV	99.58	97.32	82.76	99.32	97.35	81.98	97.12	85.39	98.70	85.52
Gabor filter [31]	99.50	97.80	92.20	Nil	97.90	92.30	97.90	94.80	97.80	94.80
BR	99.50	98.60	92.60	99.60	98.50	86.00	98.60	94.50	98.60	94.70
NTTP	99.96	99.41	94.81	99.99	99.44	94.74	99.16	95.07	99.10	95.09

**Table 2.** Maximum, minimum and average accuracies (%) along with the standard deviation for different methods using chi-square distance and nearest neighbor classifier.

		**LBP**	**CLBP_S/M/C**	**LTP(pre)**	**LTP**	**LBPV**	**NTTP**
**TC02**	Average	83.27	86.17	76.55	90.01	82.07	94.74
	Maximum	84.21	86.63	77.12	90.60	82.76	95.24
	Minimum	82.60	85.75	75.92	88.94	81.02	94.18
	Std. dev.	0.45	0.31	0.39	0.35	0.44	0.24
**TC05**	Average	83.23	86.23	74.92	90.01	81.98	94.74
	Maximum	84.30	86.82	76.08	90.85	82.83	95.51
	Minimum	82.12	85.16	73.39	89.01	81.15	93.99
	Std. dev.	0.54	0.37	0.50	0.38	0.42	0.31
**TC07**	Average	87.35	88.14	82.16	91.52	85.39	95.07
	Maximum	91.81	92.27	87.86	94.45	90.56	97.30
	Minimum	79.56	78.27	71.84	86.46	75.00	90.82
	Std. dev	2.73	2.62	3.22	1.63	2.87	1.24
**TC09**	Average	87.43	88.30	81.65	91.67	85.52	95.09
	Maximum	91.73	92.37	88.38	94.67	90.51	97.35
	Minimum	78.45	77.90	74.17	86.41	75.31	91.39
	Std. dev.	2.48	2.47	3.42	1.66	2.72	1.18

**Table 3.** Classification rates (%) of the different pattern codes using SVM as classifier.

	**LBP**	**CLBP_S/M/C**	**LTP**	**LBPV**	**NTTP**
TC02	92.32	94.00	95.89	93.81	97.90
TC09	92.4	93.31	96.16	93.01	97.62

**Table 4.** Classification rates (%) of the different coding schemes on the Brodatz dataset.

**Classifier**	**LBP**	**CLBP_S/M/C**	**LTP**	**LBPV**	**NTTP**
Chi-square and nearest neighbor	92.89	94.63	95.98	93.13	96.27
SVM	95.21	98.85	98.87	96.14	99.07
